# Deep learning and attention mechanisms to identify key genes and their implications for the origin of insect wings

**DOI:** 10.1038/s41598-026-49441-y

**Published:** 2026-05-06

**Authors:** Fangrong Liu, Yong Cao, Songping Qian, Xingyu Tong, Junhui Liu, Jiawei Mao, Si Li, Shiyu Li, Youjie Zhao

**Affiliations:** 1 College of Big Data and Intelligent Engineering, Southwest Forestry University, Kunming, 650224 China; 2 Institute of Zoology, Chinese Academy of Sciences, Beijing, 100101 China

**Keywords:** Insect evolution, Wings origin, Gene identification, Wing genes, Deep learning, Attention mechanisms, Ecology, Ecology, Evolution, Genetics, Zoology

## Abstract

Wings are a key trait innovation in the evolutionary history of insects, and contributes to the largest diversity of animals on the planet. However, we still have an incomplete understanding of the functional changes in genes behind this diversification. Insect, Malacostraca and Chelicerata species originated as primitive arthropods during the Cambrian period. Malacostraca as the ancestral taxa of winged insects, are key to understanding this radiation. Here, a deep learning (DL) model for wing genes identification (DeepWG) based on bidirectional long short-term memory (BiLSTM) and attention mechanism (AM) was constructed based on the protein sequences of 119 species. DeepWG demonstrated a strong potential in mining key genes of insect wings, achieving an accuracy rate of 97.3% on the test set. Our research found that the 351 key genes identified by DeepWG and their orthologs exhibit transcriptional similarity in wing and gill tissues, providing molecular evidence consistent with the Ttracheal gill theory of insect wing origin. This study not only proposes a new method for identifying key genes, but also lays the foundation for genetic studies of key evolutionary adaptations in winged insects.

**Supplementary Information:**

The online version contains supplementary material available at 10.1038/s41598-026-49441-y.

## Introduction

Insects are the largest group of animals on earth in terms of the number of individuals, originating in the Early Ordovician period 479 million years ago^1^. Winged insects appeared in the early Devonian period of the Paleozoic Era (about 420 million years ago), and the current prevailing view is that winged insects evolved from wingless insects, a process from scratch^3^. The ancestors of insects are considered to be crustaceans of the phylum Arthropoda^4^. Wings are a key trait innovation during the evolutionary history of insects, and they play a crucial role in their survival, reproduction, and the stability and diversity of ecosystems^5^. However, despite the critical importance of wings to insects, controversy remains about their origin and evolution^6^. The Tracheal gill theory suggests that the ancestors of insects were aquatic and breathed through tracheal gills, which evolved into wings when they became terrestrial^7^. The Paranotal theory suggests that the upper basal segment of the appendages of insects united with the body wall and together with the sub-basal segment formed the lateral plate of insects, and that the exopod of the respiratory upper limb segments was enlarged to form the proto-wing, which had the ability to glide, and that the basal part of the pro-wing diverged into the corresponding joints to evolve into the wings^8^. The Pleuron theory suggests that insect wings evolved from the lateral dorsal lobes of the thorax^9^. Recent molecular biology research has focused on identifying sequence homologous genes associated with wings in wingless segments, offering a new perspective on the long-standing debate over the origin of insect wings^10^. Some research findings suggest that wing formation may have originated from a “dual origin” mechanism, where two types of origin tissues evolved through the fusion of these two structures during the evolutionary process^11,12^. By comparing homologous genes between different species using methods such as deep learning (DL) we can infer the key trait genes that may have existed in the common ancestor of these species, thereby providing crucial information for understanding the mechanism of insect wing formation.

Since entering the molecular era, high-throughput techniques such as RNA-seq, ATAC-seq, and scRNA-seq have been widely used to validate the three major wing origin hypotheses at the molecular level. The molecular evidence for the Tracheal gill theory has mainly come from aquatic species, and Almudi et al. found in the *Cloeon dipterum*(*C. dipterum*) that there is a transcriptomic similarity between gills and transcriptome similarity between wings, suggesting a common genetic program between them^13^. Functional experiments in terrestrial insects have provided developmental evidence consistent with the Paranotal theory. Clark-Hachtel and Tomoyasu showed that knockdown of apterous (*ap*) and vestigial *(vg*) genes in *Tribolium castaneum* resulted in the absence of wing buds^14^. Previous studies have shown that nubbin (*nub*) is expressed in both dorsal and lateral regions during appendage development, supporting the idea that insect wings may derive from contributions of both tissues^15^. In addition, Fang et al. knocked down the Hox gene *Antp* in *Bombyx mori (B.mori)* by CRISPR-Cas9 editing, resulting in an abnormal wing phenotype, suggesting that *Antp* is required for wing development in insects^16^. However, although these studies have shown many genes that influence wing origin and evolution, the functions of these genes have not been systematically elucidated in larger-scale insect taxa. Insect wing origin and evolution involves a complex synergy of many genes, and the systematic identification of candidate genes with high contributions to wing evolution on the genome remains an urgent challenge.

With the increase in sequencing depth and species coverage, data-driven machine learning is gradually being used to overcome the above bottlenecks. Random forests have been widely applied to predict enhancer elements from genomic^17^, and support vector machines models have been used to discriminate gene expression patterns^18^. However, these models still rely on manual feature engineering when dealing with hundreds of thousands of features, with limited generalization and interpretability. In recent years, deep learning (DL) combined with attention mechanisms (AM) has become a cutting-edge direction for parsing complex traits^19,20^. Recurrent Neural Networks and Convolutional Neural Networks can automatically extract upstream and downstream cis-element features from DNA sequences^21^. Graphic Network can learn node importance in protein-interaction networks^22^. Transformer models have significantly improved recall in mammalian organ development and plant leaf morphogenesis by coupling multiple heads of attention to simultaneously focus on distant cis-regulation and temporal expression^23^. However, these models have yet to be specifically trained and validated for the ancient innovative trait of insect wings. Therefore, DL and AM are gradually replacing traditional rule-driven approaches in modeling complex trait formation mechanisms as a beneficial tool for revealing the molecular mechanisms of the origin of insect wings.

In this study, we developed a framework called “DeepWG” (Deep learning model for wing genes identification), which employs deep learning to identify proteins from both wingless and winged species. Firstly, we collected protein sequences from national center for biotechnology information (NCBI) databases to construct the benchmark dataset. Subsequently, AM was introduced on the basis of Bi-directional Long Short-Term Memory (BiLSTM) to mine the wing genes. Finally, the analysis of functional enrichment and gene expression was performed. DeepWG can quickly and accurately identify insect wing genes, providing new insights into the molecular mechanisms behind the origin of insect wings.

## Materials and methods

### Data collection and wrangling

**Fig. 1:**
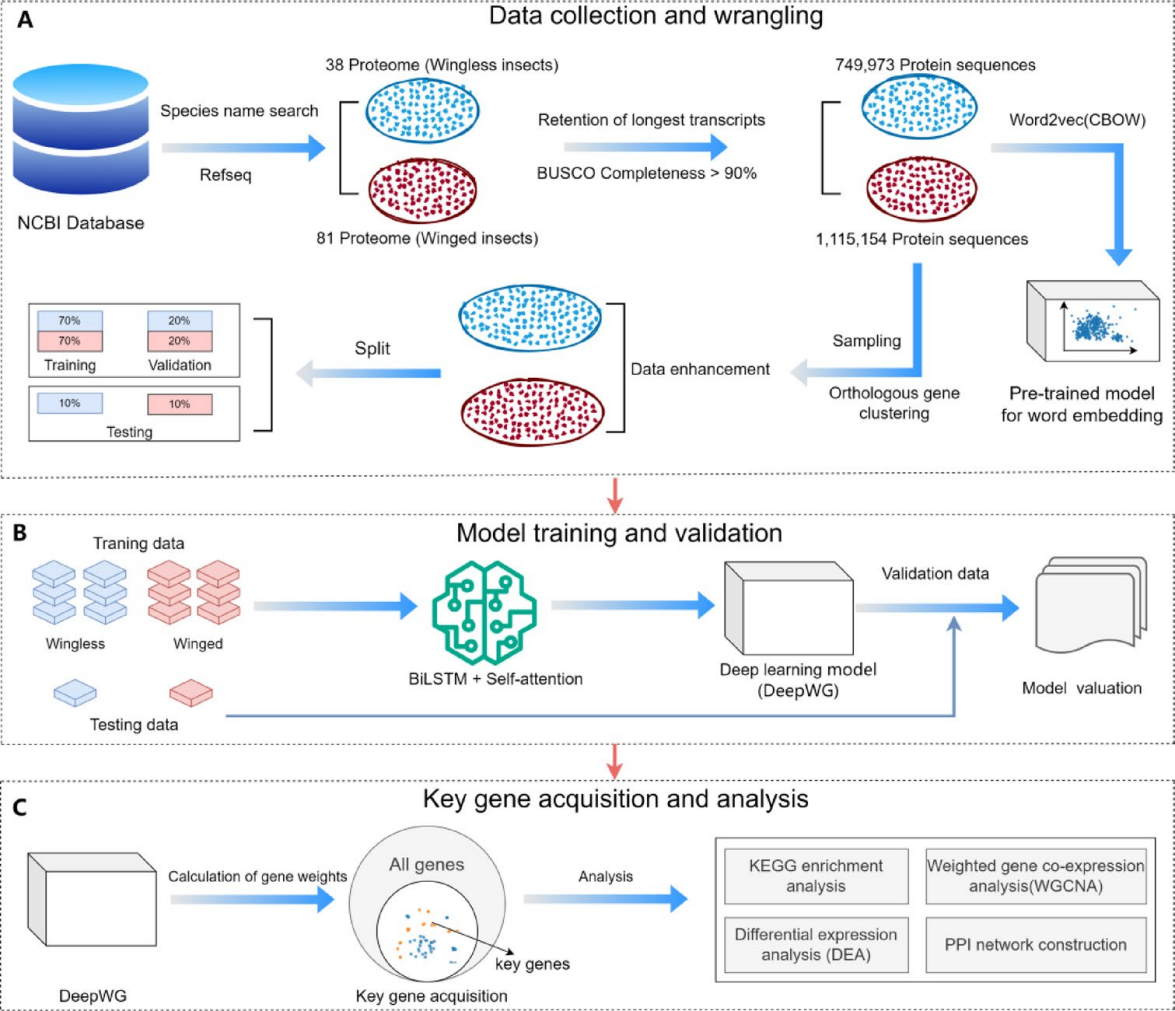
Study design overview. (**A**) Data collection and wrangling process. (**B**) Model training. (**C**) Key gene acquisition and analysis.

The first step in developing the prediction model – DeepWG – was data collection and wrangling (Fig. 1A). Protein sequences of 119 species were selected from the NCBI (https://www.ncbi.nlm.nih.gov/) database^24^, and these protein sequence were used as the constructed dataset for DeepWG (Table S1). The 119 species were categorized into 38 wingless and 81 winged based on the evolutionary characteristics of wings (Table 1), and the molecular mechanisms of key genes on the origin and evolution of insect wings were investigated by DeepWG.

The completeness of the proteomics (FASTA format) of each species was subsequently assessed using BUSCOv5^25^, and only FASTA sequence files with BUSCO scores above 90% were retained for subsequent analysis. We trained a word embedding pre-training model based on these preprocessed protein sequences using word2vec for subsequent vectorized representation of the sequences. Meanwhile, orthogonal gene clustering was performed on all protein sequences using OrthoFinder to obtain Orthogroup (orthologous gene family) across species^26^, and randomly sampled from them before each training to ensure the systematic diversity and evolutionary representation of the samples. The data-enhanced genomes were divided into training, validation and test sets in the ratio of 7:2:1.

**Table 1:**
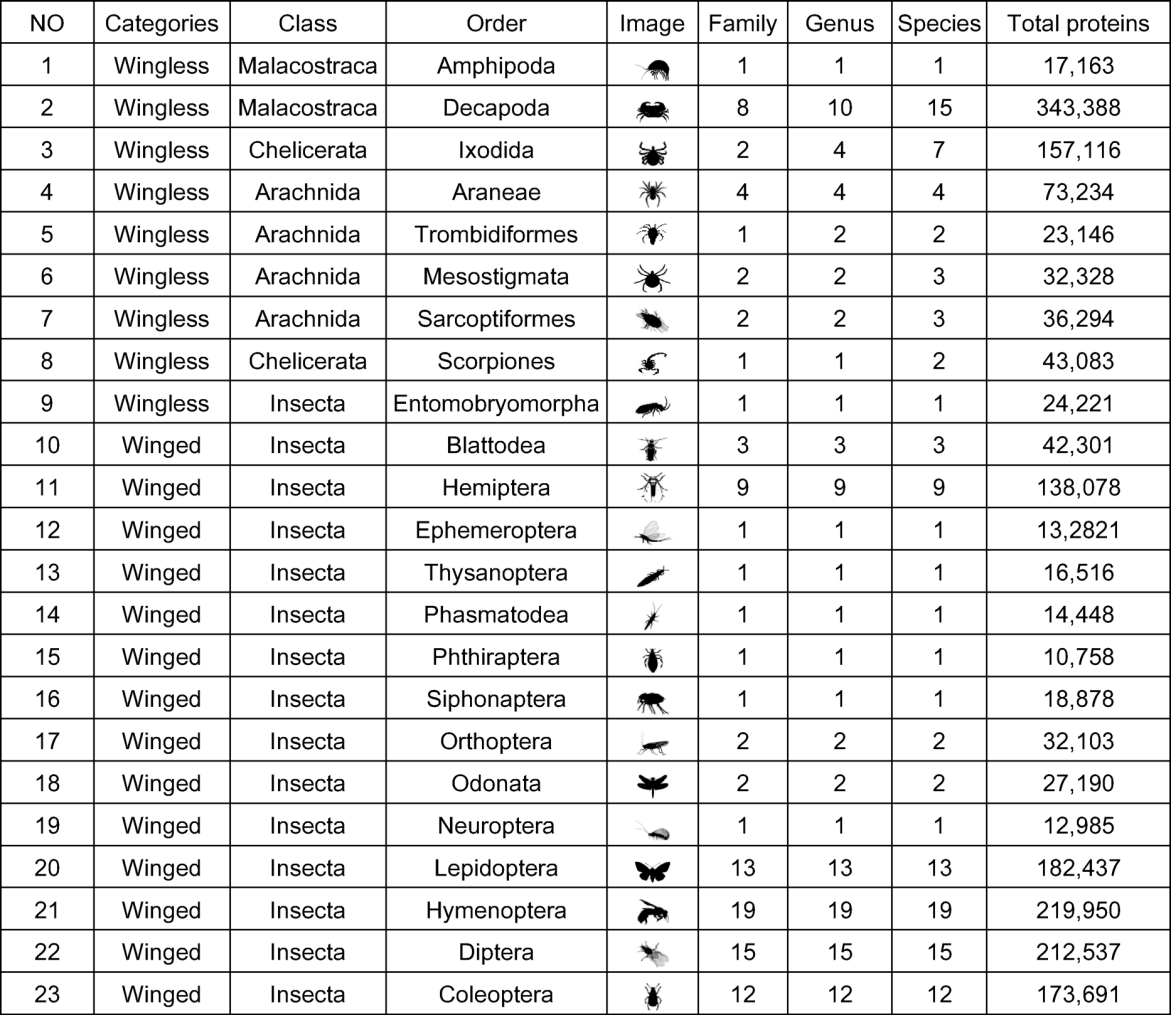
Protein sequences of 119 species from 23 orders. Images of representative taxa in the 23 orders were obtained from PhyloPic (http://phylopic.org).

### Study design overview

Our study comprised several key steps: Data collection and wrangling (Fig. 1A), Model training (Fig. 1B) and Key gene acquisition and analysis (Fig. 1C). A detailed description of data collection and wrangling is described in "Data collection and wrangling". We develop DeepWG deep learning models for key gene identification based on training data. The architectural design of DeepWG is carried out by means of bidirectional long short-term memory network (BiLSTM) and self-attention mechanism, and the performance of the model is evaluated using a test set. Finally, we use trained DeepWG to access key genes and parse their impact on the origin and evolution of insect wings.

### Model training and validation

**Fig. 2:**
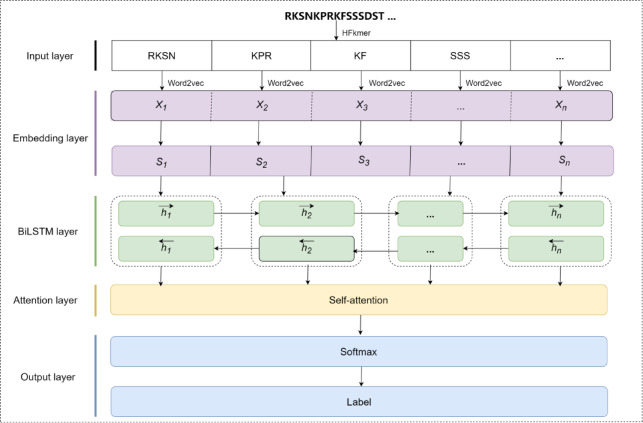
The deep learning framework of DeepWG. The DeepWG architecture includes Input layer, Embedding layer, BiLSTM layer, Attention layer and Output layer. The input protein sequence is first tokenized into words using HFkmer, and the embedding dimension of word2vec is 150. *Xn* denotes the *nth* word in this sequence and *Sn* denotes the *nth* sequence in this species.

We consider the task of protein identification in wingless/winged species as a binary classification problem. Thus, we have designed a deep learning framework called “DeepWG” (Fig. 2). DeepWG is a deep learning framework containing a BiLSTM equipped with a self-attention module to efficiently learn higher-order feature representations of all proteins. DeepWG was developed based on PyTorch, and we employed the ‘Adam’ optimizer^27^ with a learning rate of 0.001. DeepWG is able to automatically learn the feature representation of protein sequences, eliminating the need for manual computation or extraction of features by humans. We use raw protein sequences as input and employ High frequency kmer (HFkmer) for disambiguation (the detailed process is shown in Figure S1), and then the embedding layer encodes the initial representation of the input sequences. Deep learning methods are able to learn high-quality feature representations directly from sequence embeddings, rather than relying on manually filtered features. Therefore, we use sequence embedding method to encode the input sequences. Due to the differences in the length of the protein sequences, during the training process, we make the sequences of the same length by padding the end of the sequence with zeros (zeros represent any unknown amino acid type).

#### HFkmer and embedding vectors

Biological language has a one-to-one relationship with natural language, in which a single amino acid corresponds to a word in a sentence, and the protein-coding genes of a single species correspond to a sentence composed of multiple words. However, the selective evolution of genes is often reflected by small differences in amino acid sequences, and directly treating amino acids as words not only fails to effectively reveal the molecular mechanism of the origin and evolution of insect wings, but also results in lengthy information. Mao et al. found in the genome of Lepidoptera that the size of the word list increases exponentially with the k value, and the k value directly affects the size of the word list and the composition of the final word list, which in turn affects the final result of vocabulary hierarchy construction^28^. Therefore, the length of kmer used in this study is consistent with the study of Mao et al. i.e. k = 3. Finally, we filtered out the HFkmer based on kmer of 3, and then based on this HFkmer, all protein sequences were subclassified.

Word embedding is an important technique in the field of natural language processing. It overcomes the limitations of traditional one-hot coding methods and effectively captures semantic and syntactic relationships by mapping words into a low-dimensional continuous vector space. In this paper, word embedding pre-training is performed using the CBOW algorithm on the data represented by the word hierarchy to obtain a sequence vector representation with an embedding dimension of 150. The internal words of each protein sequence are replaced with the word vector representation obtained from the pre-trained model, which is then converted into a feature matrix by concatenating all the word embedding vectors in that protein sequence. Similarly, the corresponding feature matrices of each protein sequence were concatenated to obtain a complete vector representation of all protein sequences in a species. For each target word $\:{w}_{t}$, its surrounding context words within a window of size $\:c$ are mapped to embedding vectors $\:{v}_{{w}_{t+j}}\in\:{\mathbb{R}}^{d}$. The context representation is computed as the average of the surrounding word vectors:

1 $$\:{v}_{context}=\frac{1}{2c}\sum\:_{-c\le\:j\le\:c,j\ne\:0}{v}_{{w}_{t}+j}$$

where $\:c$ denotes the context window size, $\:j$ denotes the relative position index within the context window and $\:d$ is the embedding dimension.

#### The attention mechanism

Based on the AM, protein sequences can be viewed as moving from words (amino acids) to sentences (genes) and documesnts, so that models of the attention mechanism can be designed accordingly. The DeepWG for processing protein sequences of insects was able to identify important regions, and the amino acids in each sequence were then processed by the BiLSTM. LSTM is a variant of recurrent neural network (RNN), which consists of three gating mechanisms, namely, forgetting gate, input gate and output gate, and its detailed computational procedure is shown in Eqs. (2)-(7).

2 $$f_{t} = \sigma \:\left( {W_{f} \cdot \:\left[ {h_{{t - 1}} ,x_{t} } \right] + b_{f} } \right)$$

3 $$i_{t} = \sigma \:\left( {W_{i} \cdot \:\left[ {h_{{t - 1}} ,x_{t} } \right] + b_{i} } \right)$$

4 $$o_{t} = \sigma \:\left( {W_{o} \cdot \:\left[ {h_{{t - 1}} ,x_{t} } \right] + b_{o} } \right)$$

5 $$\mathop c\limits^{\sim } _{t} = \tanh \left( {W_{c} \cdot \:\left[ {h_{{t - 1}} ,x_{t} } \right] + b_{c} } \right)$$

6 $$c_{t} = f_{t} \odot \:c_{{t - 1}} + i_{t} \odot \:\mathop c\limits^{\sim } _{t}$$

7 $$h_{t} = o_{t} \odot \:\tanh \left( {c_{t} } \right)$$

where$\:{f}_{t}$, $\:{i}_{t}$ and $\:{o}_{t}$ denote the forgetting gate, input gate and output gate respectively. $\:{x}_{t}\:$represents the input at the current time step, and $\:{h}_{t}$ represents the output at the current time step. $\:\sigma\:$ represents the Sigmoid activation function, with an output range of 0 to 1. $\:W,b$ are the weight matrix and bias term to be learned, respectively, and $\:\mathrm{t}\mathrm{a}\mathrm{n}\mathrm{h}$ is also a nonlinear activation function.

After that, the internal state $\:{c}_{t}$ (also known as the memory cell) is updated by combining the $\:{f}_{t}$ and $\:{i}_{t}$, where $\:{f}_{t}$ determines how much information is forgotten from the previous time portion of the memory cell $\:{c}_{t-1}$, and $\:{i}_{t}$ determines how much information is input from the candidate memory cell $\:{\stackrel{\sim}{c}}_{t}$ at the current time step. Finally, the internal state $\:{c}_{t}$ is used as the output for the current time step by combining it with the output gate $\:{o}_{t}$. BiLSTM consists of forward LSTM and backward LSTM. In bi-directional LSTM, there are two directions of hidden states, the forward hidden state $\:{\overrightarrow{h}}_{i}$ and the backward hidden state $\:{\overleftarrow{h}}_{i}$, as shown in Eqs. (8)-(10):

8 $$\:{\overrightarrow{h}}_{i}=\overrightarrow{\mathrm{L}\mathrm{S}\mathrm{T}\mathrm{M}}\left({\mathrm{s}}_{i},{\overrightarrow{\mathrm{h}}}_{\mathrm{i}-1}\right)$$

9 $$\:{\overleftarrow{h}}_{i}=\overleftarrow{\mathrm{L}\mathrm{S}\mathrm{T}\mathrm{M}}({\mathrm{s}}_{\mathrm{i}},{\overleftarrow{\mathrm{h}}}_{\mathrm{i}+1})$$

10 $$\:{h}_{i}=[{\overrightarrow{h}}_{i},{\overleftarrow{h}}_{i}]$$

where $\:{\overrightarrow{h}}_{i}$ and $\:{\overleftarrow{h}}_{i}$ denote the forward and backward implicit variables of $\:{\overrightarrow{\mathrm{h}}}_{\mathrm{i}-1}$ and $\:{\overleftarrow{\mathrm{h}}}_{\mathrm{i}+1}$, respectively, for the ith sentence in the text. In this study, AM is added to BiLSTM to dynamically adjust the weights of different parts of the input sequences, which enables the model to process the sequences more flexibly, and the attention scores are calculated as shown in Eq. (11).

11 $$\:{e}_{ij}=\frac{\left(Q{W}_{Q}\right){\left(K{W}_{K}\right)}^{T}}{\sqrt{{d}_{k}}}$$

where $\:Q$ is the query matrix, $\:K$ is the key matrix, $\:{W}_{Q}$ is the query weight moment, and $\:{W}_{K}$ the key weight matrix. $\:{d}_{k}$ is the dimension of the key vector, which is used for scaling dot product. Attention weight $\:{a}_{ij}$ is the result of score $\:{e}_{ij}$ after softmax function, which represents the attention weight of the ith query vector to the jth key vector. The attentional weight $\:{a}_{ij}$ is calculated as shown in Eq. (12) and finally the value vectors of the sentence are weighted and summed using the attentional weights as shown in Eq. (13).

12 $$\:{a}_{ij}=softmax\left({e}_{ij}\right)=\frac{exp\left({e}_{ij}\right)}{\sum\:_{k=1}^{n}exp\left({e}_{ik}\right)}$$

13 $$\:c_{i} = \sum {\:_{{j = 1}}^{n} } a_{{ij}} V_{j}$$

#### Performance evaluation

DeepWG is quantitatively evaluated using the following metrics: accuracy (Acc), Precision (Pre), Recall (Re), F1 score (F1), and area under the receiver operating characteristic (ROC) curve (AUC). Their definitions are as follows:

14 $$Acc = \frac{{TP + TN}}{{TP + TN + FP + FN}}$$

15 $$Pre = \frac{{{\mathrm{TP}}}}{{{\mathrm{TP}} + {\mathrm{FP}}}}$$

16 $$\mathrm{Re} = \frac{{TP}}{{TP + FN}}$$

17 $$F1 = \frac{{2 \times \:{\mathrm{Precision}} \times \:{\mathrm{Recall}}}}{{{\mathrm{Precision}} + {\mathrm{Recall}}}}$$

where *TP* (True Positive) denotes the number of positive samples correctly classified, *TN* (True Negative) denotes the number of negative samples correctly classified, FP (False Positive) i.e., the number of misclassified as actual samples, and *FN* (False Negative) denotes the number of actual labels not predicted by the model in classification. The area under the curve (AUC) score was calculated by measuring the area under receiver operating characteristic (ROC) curve to assesses the model’s ability in distinguish between different classes.

### Relative expression level

To obtain transcriptome data from different tissue of the *Drosophila melanogaster* (*D. melanogaster)*, *Cloeon dipterum* (*C. dipterum*), and *Penaeus vannamei* (*P. vannamei)*. we searched the NCBI’s Sequence Read Archive (SRA) database (https://www.ncbi.nlm.nih.gov/sra) using the species name and tissue name as keywords to obtain the corresponding RNA-seq SRA login numbers. Eventually, we downloaded transcriptome data for a total of 51 samples (Table S2). For the candidate genes identified by DeepWG that are related to the origin and evolution of insect wings, we used StringTie v2.2.1 for transcript assembly and expression estimation of the raw data^29^.

To achieve comparable expression levels, we normalized transcript abundance to transcripts per million (TPM), and the normalization process consisted of the following steps: firstly, the read coverage of each transcript was calculated, then the raw counts were corrected according to the transcript lengths, and finally the expression was scaled by combining with the total sequencing depth of each sample to obtain a uniform abundance metric. Soft clustering analysis was conducted using the Mfuzz software based on normalized read counts^30^. Genes were clustered according to their expression patterns across different tissues to identify tissue-specific expression modules.

### Weighted gene co-expression network analysis

Weighted gene co-expression network analysis (WGCNA) is a systems biology approach aimed at identifying groups (modules) of genes with similar expression patterns from to reveal their synergistic roles in common biological processes^31^. Its analysis process typically involves constructing gene co-expression networks, performing module delineation, associating modules with phenotypic features, etc., evaluating inter-module relationships, and screening for potential key genes within a target module. We used WGCNA in R (Version 1.71) to explore modules associated with insect wings. We input an expression matrix consisting of 13,962 genes across *D. melanogaster* into WGCNA. The min Module Size parameter indicates the minimum number of genes in a module, and the smaller the value, the smaller the retained modules. Merge Cut Height indicates the distance at which similar modules are merged, and the smaller the value, the lower the merging possibility and the more modules are retained. The smaller the value, the smaller the possibility of merging and the more modules will be retained. The min Model Size is set to 50 and merge Cut Height is set to 0.25. The default settings are consistent with the WGCNA tutorial (https://rpubs.com/natmurad/WGCNA) and relevant published studies^32,33^.

### Construction of the PPI network

Protein-protein interactions (PPIs) are fundamental elements of cellular biochemical networks and are essential for regulating cellular functions and signaling pathways^34^. Understanding the complex relationships among multiple proteins through systematic analysis can help to gain insights into biological signaling response mechanisms and energy-matter metabolism under specific physiological conditions, as well as elucidate the functional interactions among proteins^35^. To analyze PPIs, we entered the shared genes identified in our study into the STRING database (version 12.0, https://string-db.org/)^36^. We focused on retaining PPIs that were not independent nodes and had a composite score > 0.4 to ensure the relevance and reliability of the interactions based on the recommended results. The generated PPI network was then visualized using Cytoscape software (version 3.10.3)^37^. Within the network, node boundary thickness corresponds to connectivity, with thicker boundaries indicating higher interactions. Similarly, edge thicknesses represent interaction scores, with thicker lines indicating higher correlation.

### KEGG enrichment analysis

KEGG pathway enrichment analysis was performed to identify significantly enriched biological pathways associated with the identified gene sets. Functional enrichment analysis of Kyoto Encyclopedia of Genes and Genomes (KEGG) pathways was performed using the R package “clusterProfiler” (v4.16.0)^38^. A path with p.value < 0.05 was considered to be a result with significant enrichment.

## Results

### Data wrangling results

**Fig. 3:**
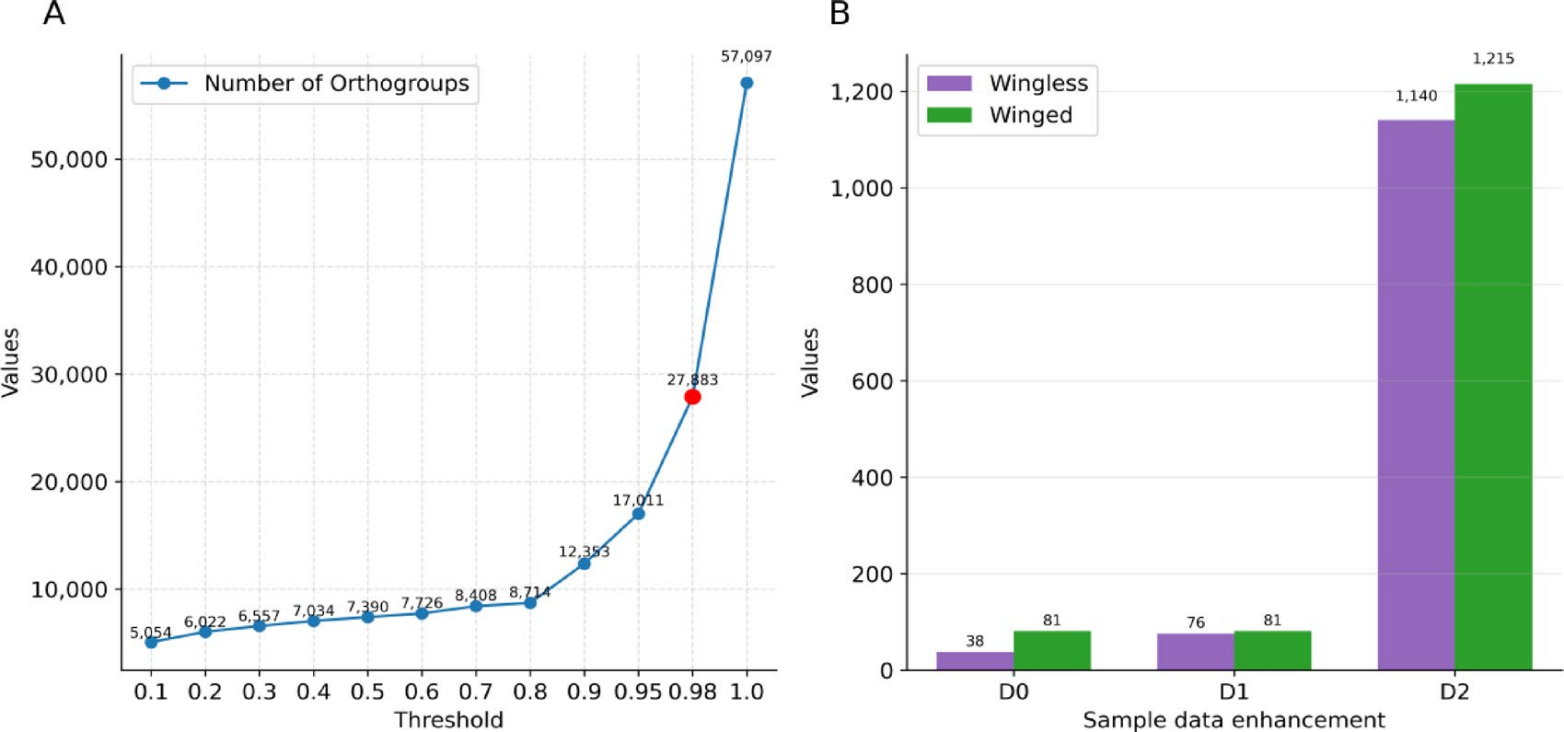
Results of data collection and wrangling. (**A**) Orthogroups’ length selection strategy. When the length is 27,883, it can cover 98% of species. A larger threshold value means that there are fewer species with orthologs genes in each Orthogroup. (**B**) Sample size after data enhancement. D0 represents the original sample, D1 represents the dataset of hemimetabolous insect samples after enhancement with the original sample data, whereas D2 represents the dataset after enhancement based on orthologous gene sampling.

Using proteomes of 119 species from wingless and winged insects were obtained from NCBI. Completeness assessment showed that the BUSCO values of these proteomes were all above 90%, with an average of 96.9% (Table S1). From these 119 species, 1,865,127 proteins with the longest transcripts were obtained (Table S1). Using the HFkmer disambiguation, 6,244 kmer were extracted from 1,865,127 protein sequences. To meet the sample size requirement for DeepWG, we modeled and extended the samples. 57,097 Orthogroups were clustered using Orthofinder, and 27,883 Orthogroups that were present in more than 98% of the species were selected for the construction of the DeepWG (Fig. 3A). Classification based on 27,883 Orthogroups, 1,140 wingless samples and 1,215 winged samples from 119 species (Fig. 3B). These 2355 samples were divided into 1,648 training samples, 471 validation samples, and 236 test samples for DeepWG training in a ratio of 7:2:1. The protein sequences of all 2,355 samples were quantified by 6,244 words vectors and used to construct DeepWG.

### DeepWG training and performance evaluation

**Fig. 4:**
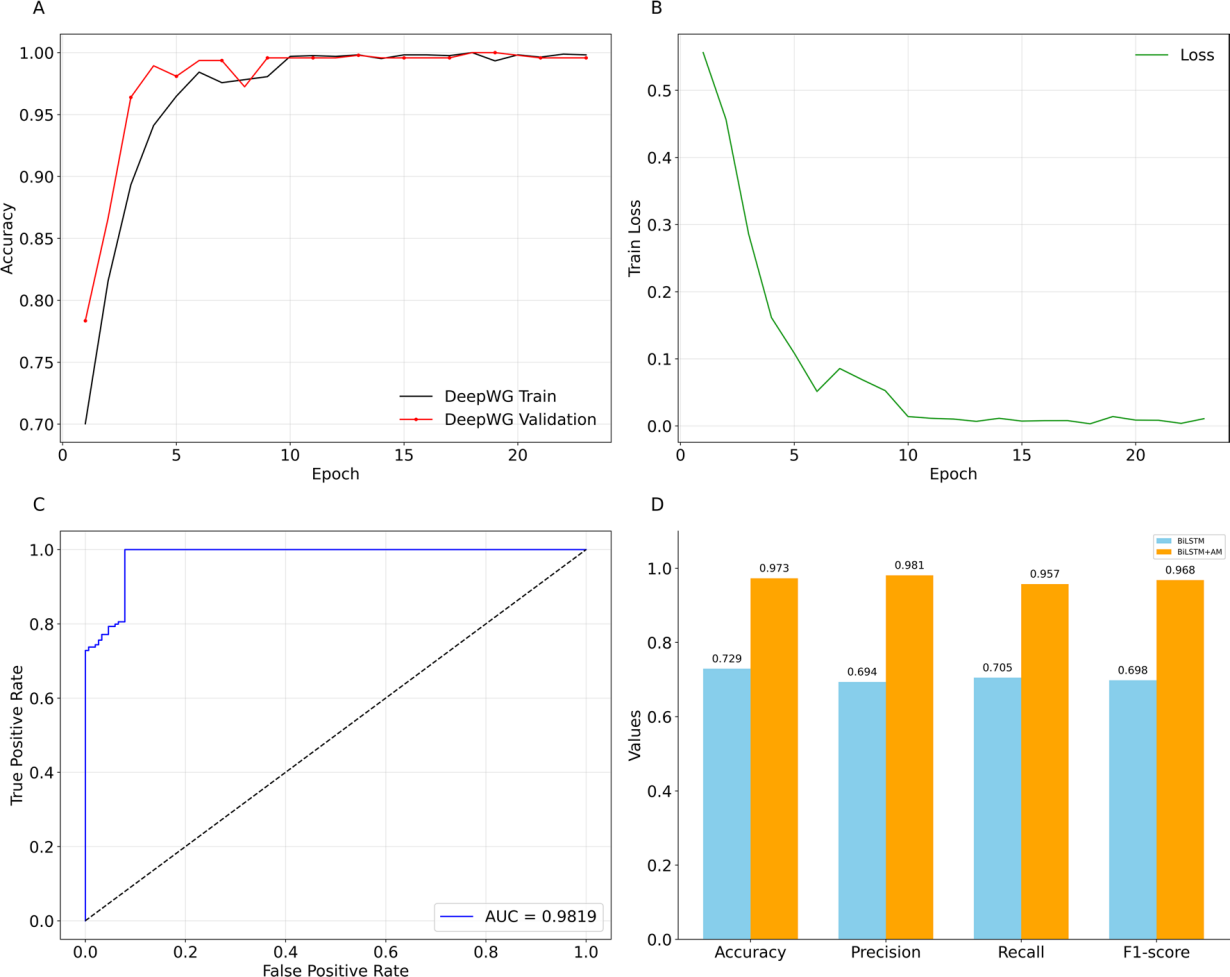
DeepWG training and performance evaluation. (**A**) Accuracy curves for DeepWG training and validation. (**B**) DeepWG training loss curve. (**C**) DeepWG ROC curve. (**D**) Comparison results of the BiLSTM and DeepWG models in terms of accuracy, precision, recall, and F1-score metrics. The blue bars represent BiLSTM, and the orange bars represent DeepWG. The values represent the scores for the corresponding metrics.

During the training process of DeepWG, we adopt the “dropout” and “early stopping” strategies to avoid overfitting, and we also continuously monitor the changes of the model’s loss and accuracy through the training and validation sets. Figure 4 shows the trend of loss and accuracy during DeepWG training. The results show that the training accuracy and training loss of DeepWG show a good convergence trend with training iterations (Fig. 4A, B). As shown in Fig. 4C, when tested on the 236 test samples, DeepWG based on the AM achieved an AUC value of 0.9819. To validate the effectiveness of the AM in enhancing the performance of the BiLSTM, we compared the performance of the BiLSTM and DeepWG models trained on the same dataset across 4 commonly used classification performance metrics (Fig. 4D). The results show that the DeepWG model significantly outperforms the BiLSTM model in terms of Accuracy (0.973), Precision (0.981), Recall (0.957), and F1-score (0.968), all of which were significantly higher than those of BiLSTM (0.729, 0.694, 0.705, and 0.698, respectively). This indicates that the introduction of the AM effectively enhances the model’s ability to capture key information, thereby achieving a significant advantage in overall performance. These metrics highlight the reliability of DeepWG in accurately distinguishing between wingless and winged insect samples, reflecting its ability to identify genes associated with wings.

### Feature representations learned by DeepWG

**Fig. 5:**
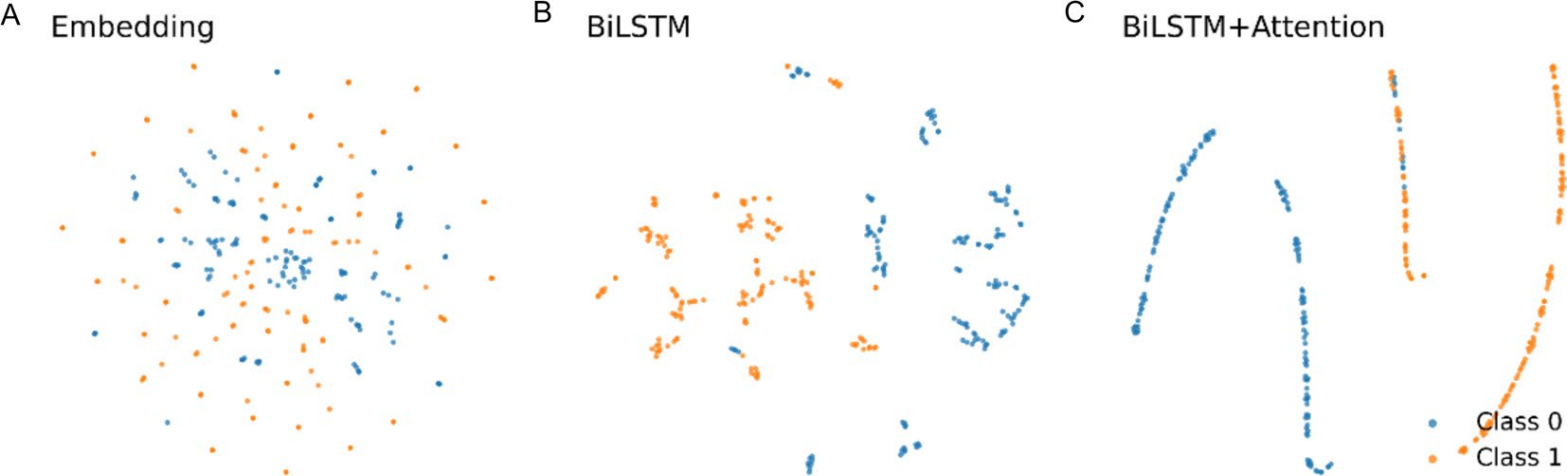
Feature distribution visualizations using t-SNE plots of different layers in DeepWG. (**A**) Sequence embedding. (**B**) The BiLSTM layer. (**C**) The attention layer in DeepWG. The dots with different colors in the figure represent different types of proteins. class 0 denotes protein sequences of wingless insects and class1 denotes protein sequences of winged insects.

We evaluated the effectiveness of feature representations learned by the DeepWG in distinguishing between wingless and winged insect protein sequences. To do this, we visualised the representations in a t-SNE plot of features learned by the embedding layer, BiLSTM layer, and the attention layer. Figure 5A reveals that the protein sequence embeddings learned in the embedding layer cannot be used to discriminate different samples because the dots show an aggregate state. This relates to different wingless and winged insect proteins having relatively high sequence similarity. Surprisingly, the features learned by the BiLSTM layer showed considerable potential to classify these proteins (Fig. 5B), but differentiation was not absolute. Further down the attention layer, the network generated better results (Fig. 5C). The t-SNE plots demonstrate that DeepWG can learn effective feature representations from the simple sequence embedding of the input sequences to accurately discriminate wingless and winged insect protein sequences.

### Weight acquisition and identification of modules genes

**Fig. 6:**
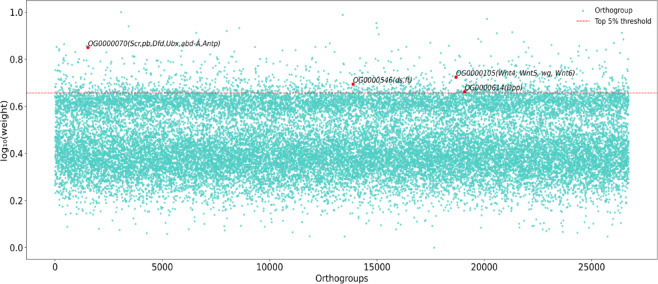
Weight distribution of Orthogroups computed by DeepWG. Top 5% threshold indicates the top 5% Orthogroups with higher weights.

The weights of 27,883 Orthogroups were obtained from the AM of DeepWG (Table S3), these values are mainly distributed between 0 and 1 (Fig. 6). Based on the DeepWG calculation results, the top 5% of 27,883 orthogonal groups were selected, and the genes in these high-weight Orthogroups were considered candidate genes for the evolution of insect wing. The results showed that a total of 3,872 genes were distributed in 1,394 Orthogroups. These key genes include *vestigial* (*vg*), *dachshund* (*ds*), *fat* (*ft*), *wingless* (*wg*), *engrailed* (*en*), *nubbin* (*nub*), *expanded* (*ex*), *decapentaplegic* (*dpp*), *frizzled*(*fz*), *epidermal growth factor receptor* (*Egfr*) and others.

**Fig. 7:**
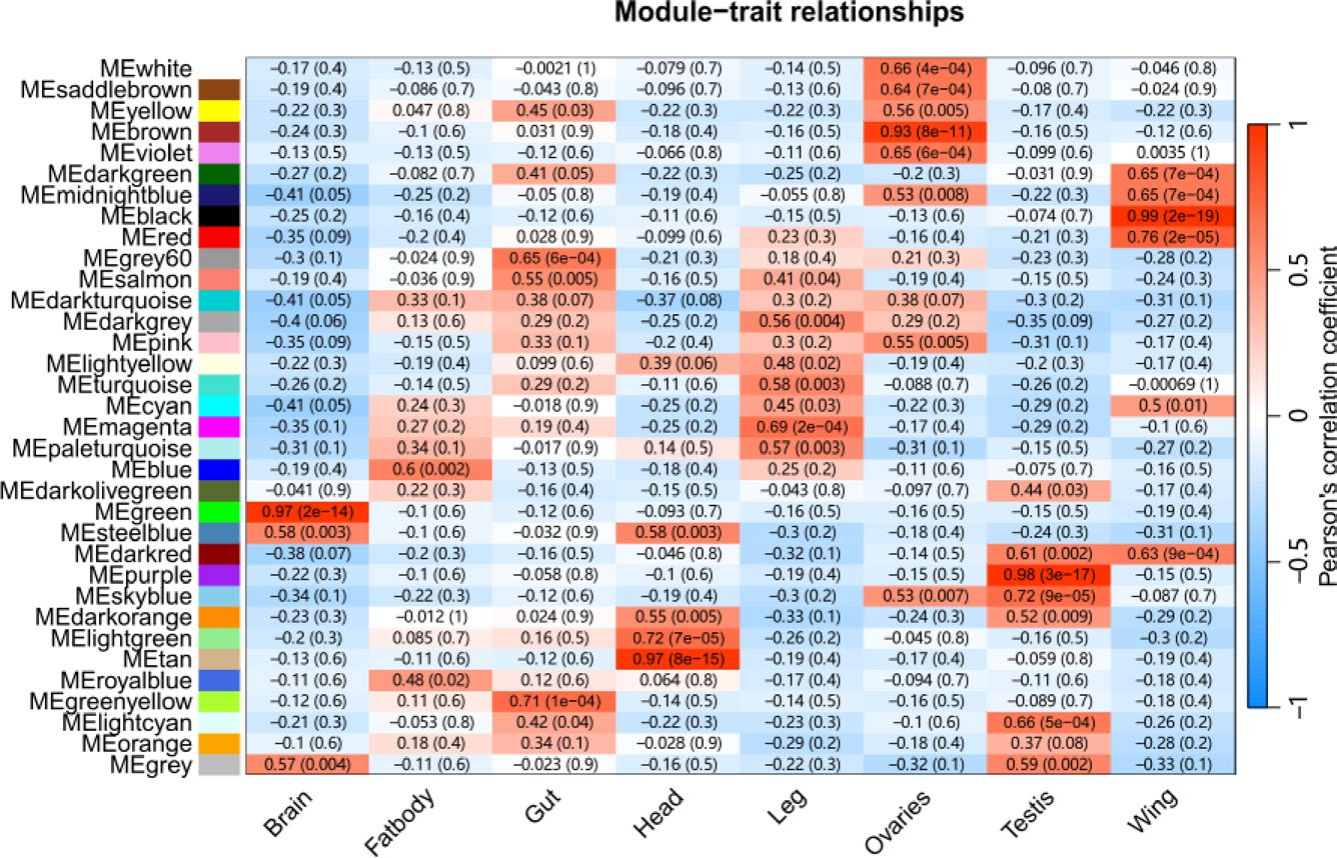
Heat map of the relationship between modules and wing trait characteristics. 17 modules included: black (599 genes), blue (1,566 genes), brown (1,050 genes), cyan (254 genes), darkgreen (95 genes), darkgrey (82 genes), darkolivegreen (24 genes), darkorange (70 genes), darkred (116 genes), darkturquoise (84 genes), green (651 genes), greenyellow (351 genes), grey (210 genes), grey60 (159 genes), lightcyan (210 genes), lightgreen (137 genes), lightyellow (126 genes), magenta (475 genes), midnightblue (217 genes), orange (74 genes), paleturquoise (32 genes), pink (483 genes), purple (458 genes), red (614 genes), royalblue (117 genes), saddlebrown (53 genes), salmon (265 genes), skyblue (62 genes), steelblue (36 genes), tan (324 genes), turquoise (1,940 genes), violet (30 genes), white (64 genes), and yellow (831 genes).

To verify that the wing-related genes identified by DeepWG are accurate and reliable, we constructed a co-expression network of wing-related genes using WGCNA. WGCNA groups genes into modules based on similar expression patterns and examines the correlation between these modules and specific traits. We constructed networks between genes and modules using *D. melanogaster* as a model organism while ensuring that R² > 0.85 for optimal network connectivity (Figure S4). The gene dendrogram and module color map are shown in Figure S5, where the top panel shows the gene hierarchical clustering dendrogram and the bottom panel indicates the corresponding gene modules. This combination indicates that genes in close proximity in the dendrogram are grouped into the same module. A total of 17 modules were identified (Fig. 7, Table S4), of which darkgreen (95 genes), midnightblue (217 genes), black (599 genes), red (614 genes), cyan (254 genes), and darkred (116 genes) were positively and statistically significantly correlated with the development of insect wings (P value < 0.05). Notably, 351 of the key genes identified by DeepWG overlap with genes in the 6 modules above, and these genes are critical for insect wing development (Figure S6, Table S5).

### KEGG enrichment analysis of key genes

**Fig. 8:**
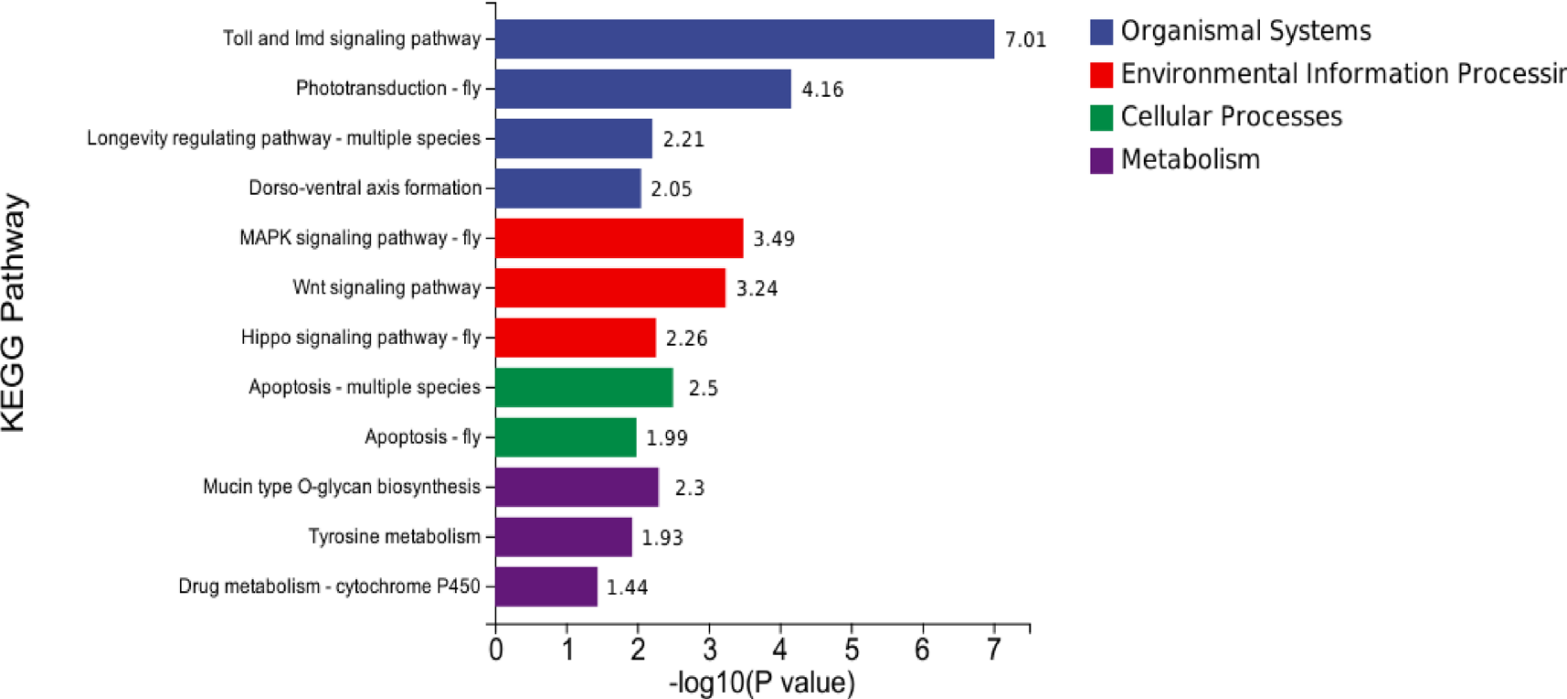
KEGG enrichment analysis results of high-weight genes. The reference species is *B. mor*i, -log10(P value) is the p value in -log base 10. These pathways were obtained via www.kegg.jp/kegg/kegg1.html, Kinkyu Laboratories has gladly granted permission.

The evolution of insect wings is of great significance to insects, and exploring the relevant pathways affecting the growth and development of insect wings will help to understand the process of wing evolution and development. To further systematically understand the functions of 3,872 genes, we annotated these genes using the KEGG database(www.kegg.jp/kegg/kegg1.html)^39,40^ in *D. melanogaster*. Analysis of the results showed that 147 genes were enriched in 16 pathways (*P* < 0.05) (Fig. 8, Table S6). Among these, 20 genes (*dpp*,* Egfr*,* bsk*, *Rac1*, *Src42A/64B*, *mts*, and *Duox* et al.) are involved in the MAPK signaling pathway – fly,18 genes (*Wnt4*,* Wnt6*,* RhoL*,* fz2*, and *pk* el al.) are involved in the Wnt signaling pathway, and 12 genes (*ft*,* ds*,* hth*,* Mad*,* rst*,* Myc*,* bsk*, and *crb* el al.) are located in the Hippo signaling pathway - fly.

**Fig. 9:**
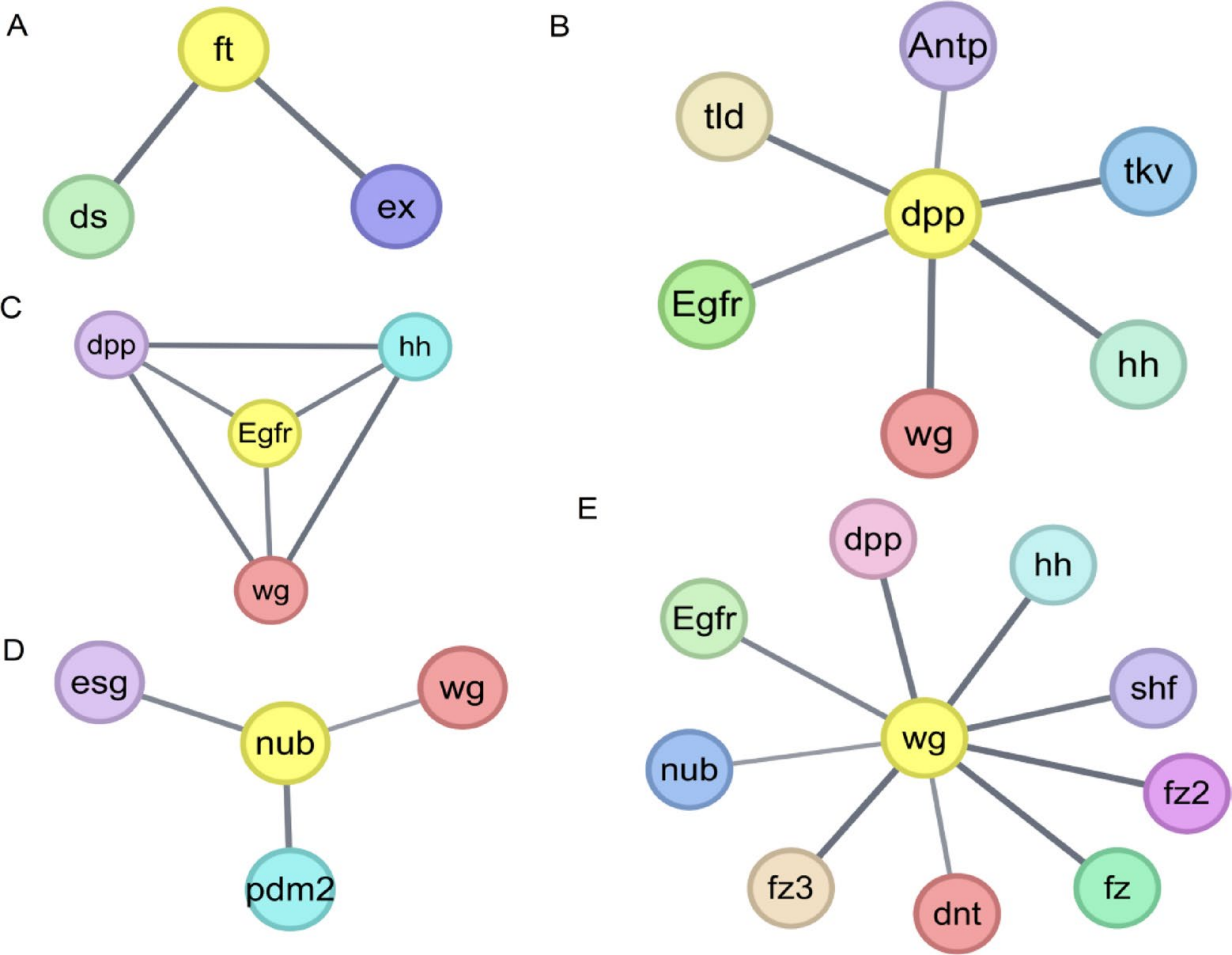
PPI network. (**A**) The PPI network of *ft*. (**B**) The PPI network of *dpp*. (**C**) The PPI network of *Egfr*. (**D**) The PPI network of *nub*. (**E**) The PPI network of *wg*. Nodes represent shared genes and edges represent interactions obtained from the STRING database. The depth of the edges represents the interaction score, the larger the combined interaction score, the thicker the edges. edges represent confidence (the thickness of the line indicates the strength of data support), the minimum required interaction score is 0.7 (high confidence).

Previous studies have shown that MAPK signaling pathway, Hippo signaling pathway - fly, and Wnt signaling pathway influence insect wing development^41–45^. Among other things, the MAPK signaling pathway is essential for wing growth. The Hippo signaling pathway plays a role in regulating cell proliferation and apoptosis to control organ size in animals. Wnt signaling mainly involves both classical and non-classical pathways, which use different ligands and receptor gene paralogs to transmit extracellular signals. Notably, we found that genes involved in these pathways were highly expressed in the wing and gills (Fig. 9). KEGG enrichment analysis showed that the key genes identified by DeepWG enriched in the MAPK signaling pathway, Hippo signaling pathway - fly, and Wnt signaling pathway were associated with insect wing development.

### PPI network and cross-tissue expression analysis of key genes

In this study, we first performed a preliminary analysis of 351 key genes using the STRING database (the minimum required interaction score is 0.7), and then used the Cytoscape tool for network visualization and refinement. Among these genes, we found that *ft* interacts with *ds* and *ex* (Fig. 9A). In addition, we found significant functional domain differences in the DNA of 24-wingless and 72-winged insects (Figure S3). In winged insects, the CADH_Y-type_LIR structural domain of *ds* was absent, which we hypothesized might be required for the development of a certain tissue in insects. Notably, we found that genes such as *dpp* (Fig. 9B), *Egfr* (Fig. 9C), and *nub* (Fig. 9D) interact with *wg* (Fig. 9E). Bone morphogenetic protein (BMP) family member *dpp* and *wg* are two morphogenetic proteins, and previous studies have shown that *wg* controls wing growth^46^.The interactions among these genes indicate that they share similar functions in the evolutionary development of insect wings.

**Fig. 10:**
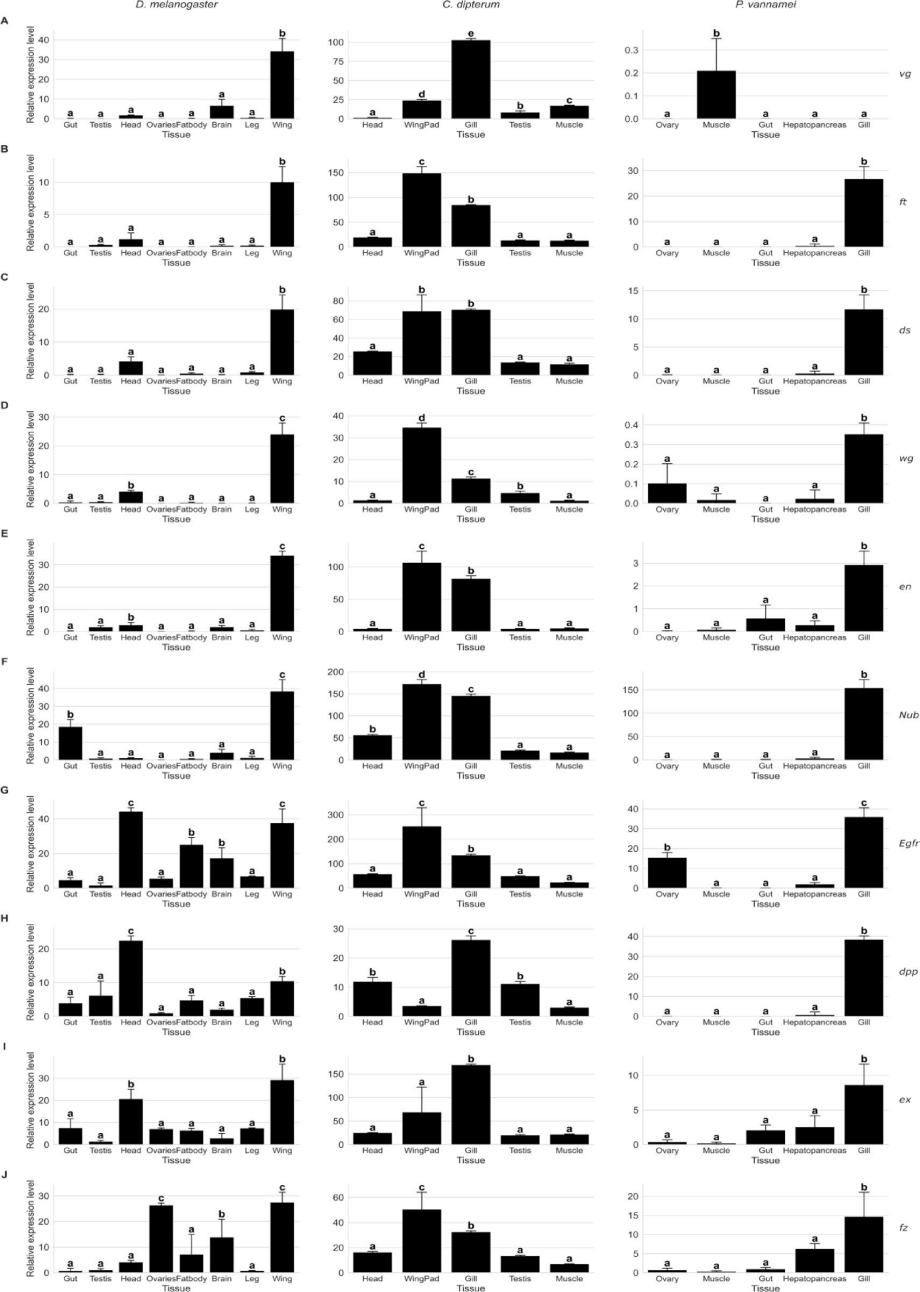
Differential expression of key genes in different tissues. Each bar is the average TPM of the stage, and the error line is the standard deviation (SD). Statistical tests were performed on log2(TPM + 1): one-way ANOVA (one-way ANOVA, log2TPM ~ Stage) was fitted to each gene and multiple comparisons (two-sided, α = 0.05) were performed with Tukey’s HSD. Different letters on the columns indicate significant differences between stages (same letter is not significant). Among them, nub, vvl and LOC113802634 belong to the immediate homologous gene family. *dpp* and *LOC113805052* are also members of the same immediate homologous gene family.

Mfuzz soft clustering analysis revealed that 351 key genes in *D. melanogaster* and their orthologs in *C. dipterum* and *P. vannamei* exhibited similar expression patterns in wing and gill tissues. In *D. melanogaster*, these genes exhibit distinct expression peaks in wing tissue (Figure S7). In *C. dipterum*, their orthologs are predominantly clustered in Clusters 1 and 2, showing elevated expression trends in wing pad and gill tissues (Figure S8). In *P. vannamei*, the relevant homologs are primarily located in Cluster 3 and exhibit specific expression in gill tissue (Figure S9). Furthermore, we examined the relative expression level of the *vg*,* ds*,* ft*,* wg*,* en*,* nub*,* Egfr*,* ex*,* fz* and *dpp* genes in different tissues of *D. melanogaster* (Table S7), *C. dipterum* (Table S8), and *P. vannamei* (Table S9). Because of their important role in *D. melanogaster* wing development, these genes were selected as wing marker genes to look for structures homologous to wings in other insects and crustaceans. The results showed that *vg*(Fig. 10A), *ft* (Fig. 10B), *ds*(Fig. 10C), *wg* (Fig. 10D), *en*(Fig. 10E), *nub*(Fig. 10F), *Egfr*(Fig. 10G), *dpp*(Fig. 10H), *ex*(Fig. 10I), and *fz(*Fig. 10J*)* genes were significantly expressed in the wings of of *D. melanogaster*, in the WingPad and gill of *C. dipterum*, and in the gill of the *P. vannamei* (in which *vg* is expressed in the muscle). This expression pattern indicates a degree of transcriptional similarity between insect wing and gill tissues.

## Discussion

Our DeepWG model, constructed based on the proteomes of 119 insects, outperforms in cross-species key gene identification, and its performance improvement is partly attributed to the integration of BiLSTM with the self-attention mechanism (Fig. 4). BiLSTM models the dependencies between sequences, while attention focuses on the importance of these sequences to the insect wings, and the two synergize to achieve accurate identification of critical genes. Although we have tried BiLSTM-based architecture in the initial stage, its performance is far below the level of DeepWG model (Figure S2). The AM not only recognizes the protein sequences in the two taxa well (Fig. 5), but also calculates the weights of these genes and enhances the interpretability of DeepWG. We also compare DeepWG’s and WGCNA’s results of wing-related genes identified in *D. melanogaster*, and despite the fact that they jointly identified only 351 genes (Table S5), the function and significance of these genes to the wing is enormous.

The question of the origin of insect wings has been centered on stimulating discussion between several hypotheses, including the Pleural origin hypothesis that the wings are considered to be a series of homologous organs of the ventral gills in the thorax^47–52^. Previous studies have shown that *vg* is a central gene determining wing fate^53^, *wg* and *dpp* form a morphogenetic signaling region that promotes wing growth^54^, *en* defines the boundary between the wing disc and the body wall^55^, *nub* is involved in the formation of the distal part of the wing blade^56^. In addition, the *dpp*-induced *Egfr* signaling pathway plays a role in regulating wing vein growth and differentiation^57^. The *ft*,* ds*, and *fz* work together to maintain cell polarity and tissue size^58,59^.

In this study, we identified transcriptional similarities between wings and gills based on 351 key genes and their orthologs in *D. melanogaster* (Holometabolous), *C. dipterum* (Hemimetabolous), and *P. vannamei* (Malacostraca), suggesting they may share common genetic programs (Figures S7, S8 and S9). Assuming that insect wings originate from the gills of their ancestral taxa or their homologous exopods, similar gene expression trends should be observed in the gills of crustaceans versus the wings/wing buds of insects. We identify a number of core genes (*vg*,* ft*,* ds*,* wg*,* en*,* ex*,* nub/vvl/LOC113802634*,* dpp/ LOC113805052*,* fz/LOC113826281* and *Egfr*) associated with wing development that may be ancestral to winged insects (Fig. 10). Our results indicate that those core genes are significantly expressed in the wings of *D. melanogaster* and wing pad and gill of *C. dipterum*, and the gill in *P. vannamei*. Thus, this evidence of gene expression across species, taxa and tissues supports the evolutionary hypothesis that insect wings originated from gills. In addition, we identified a series of previously under-reported candidate genes that may play a role in the origin and evolution of wings. These candidate genes have been narrowed down for further study of insect wings and are of great research value. Whatever the case, the transcriptomic similarity we observed between gills and wings suggests that they share a common genetic program.

Our study suggests that DeepWG is reliable in the field of key gene mining. There are a few limitations to our study. First, the origin and evolution of insect wings is a complex and multilevel regulated biological process, which is influenced by multiple factors, including epigenetic and environmental factors. Although we identified many genes associated with insect wing development, we cannot exclude the possibility that there are other functional explanations for these genes during insect evolution. Second, due to the limited availability of transcriptome samples from these insects, only the expression of these key genes in different tissues of the three model species was calculated in this study. With more and more insect genomes being sequenced and the rapid development of artificial intelligence technology, these limitations will be effectively addressed.

## Conclusions

In this study, we constructed a DeepWG model based on DL and AM by 119 species proteomes and achieved 97.3% accuracy in the test sets. DeepWG was able to accurately identify core genes related to insect wings (e.g., *vg*,* ds*,* ft*,* wg*,* en*,* nub*,* Egfr*,* dpp*,* fz* and *ex* et al.). Hierarchical visualization of t-SNE further shows that the DeepWG model extracts separable discriminative features from raw sequence embeddings from shallow to deep, reflecting the effectiveness of DeepWG in gene mining for key traits. Cross-species, cross-taxon and cross-tissue expression comparisons revealed that 351 genes and their orthologs were significantly highly expressed in the wing/wing pad of the holometabolous insect *D. melanogaster* and the hemimetabolous insect *C. dipterum*, and in the gill of the malacostraca *P. vannamei*, which represents the ancestral taxon. Therefore, the key genes identified by DeepWG and their orthologs exhibit transcriptional similarity in wing and gill tissues, supporting the gill origin hypothesis for insect wings.

## Supplementary Information

Supplementary Information 1.

## Data Availability

The 119 species proteomes used in this study are available in the Github (https://doi.org/10.6084/m9.figshare.30375376.v1) repository, all supporting data for the findings can be found in the paper and supplementary materials, and any additional information required to reanalyze the data reported in this paper is available from the lead contact upon request. Python-based experimental code was written and made publicly available on Github (https://github.com/liufangrong/DeepWG) as a reference for the experimental part of this paper.
