# Supplementary material for: Deep learning and attention mechanisms to identify key genes and their implications for the origin of insect wings
Source: Sci Rep. 2026 May 6;16:15998. doi: 10.1038/s41598-026-49441-y (PMC13197475; doi:10.1038/s41598-026-49441-y)
Supplement: Supplementary file 1 — Supplementary Information 1. [file 41598_2026_49441_MOESM1_ESM.zip › Supplementary File/Figures S1-S9.pdf]

Supplementary Figures

Deep learning and attention mechanisms to identify key genes and their implications for the origin of insect wings

This pdf file contains: Figures S1-S4:

• Figure S1: The HFkmer segmentation process.

• Figure S2: Model construction based on BiLSTM.

• Figure S3: Domain difference of *ds* genes in wingless and winged insects.

• Figure S4. Gene and module color dendrogram.

• Figure S5. Scale-free fitting exponent for soft threshold power.

• Figure S6: Number of shared genes identified by WGCNA and DeepGW.

• Figure S7: Expression patterns of 351 key genes in different tissues of *D. melanogaster*.

• Figure S8: Expression patterns of orthologs for 351 key genes across different tissues in *C. dipterum*.

• Figure S9: Expression patterns of orthologs for 351 key genes across different tissues in *P. vannamei*.

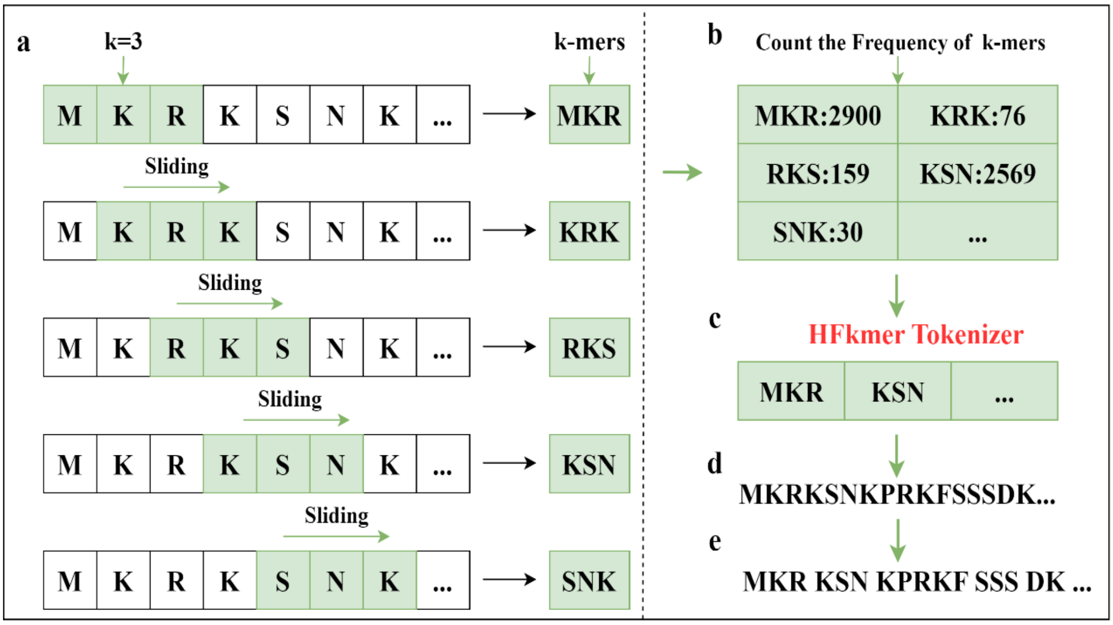

Figure S1: HFkmer Segmentation Process. (a) is the sliding window disambiguation. **(b)** is counting the frequency of each k-mers occurrence. **(c)** is the selection of high-frequency k-mers as a participle marker, called HFkmer. **(d)** represents the original genomic protein sequence. **(e)** the result of HFkmer participle.

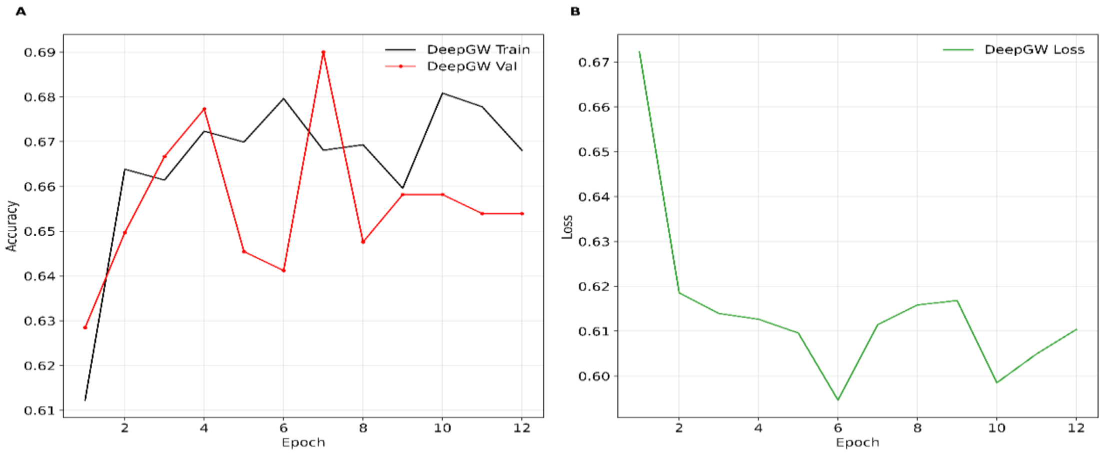

Figure S2. Model construction based on BiLSTM. (A) Accuracy curves for BiLSTM training and validation. **(B)** BiLSTM training loss curve.

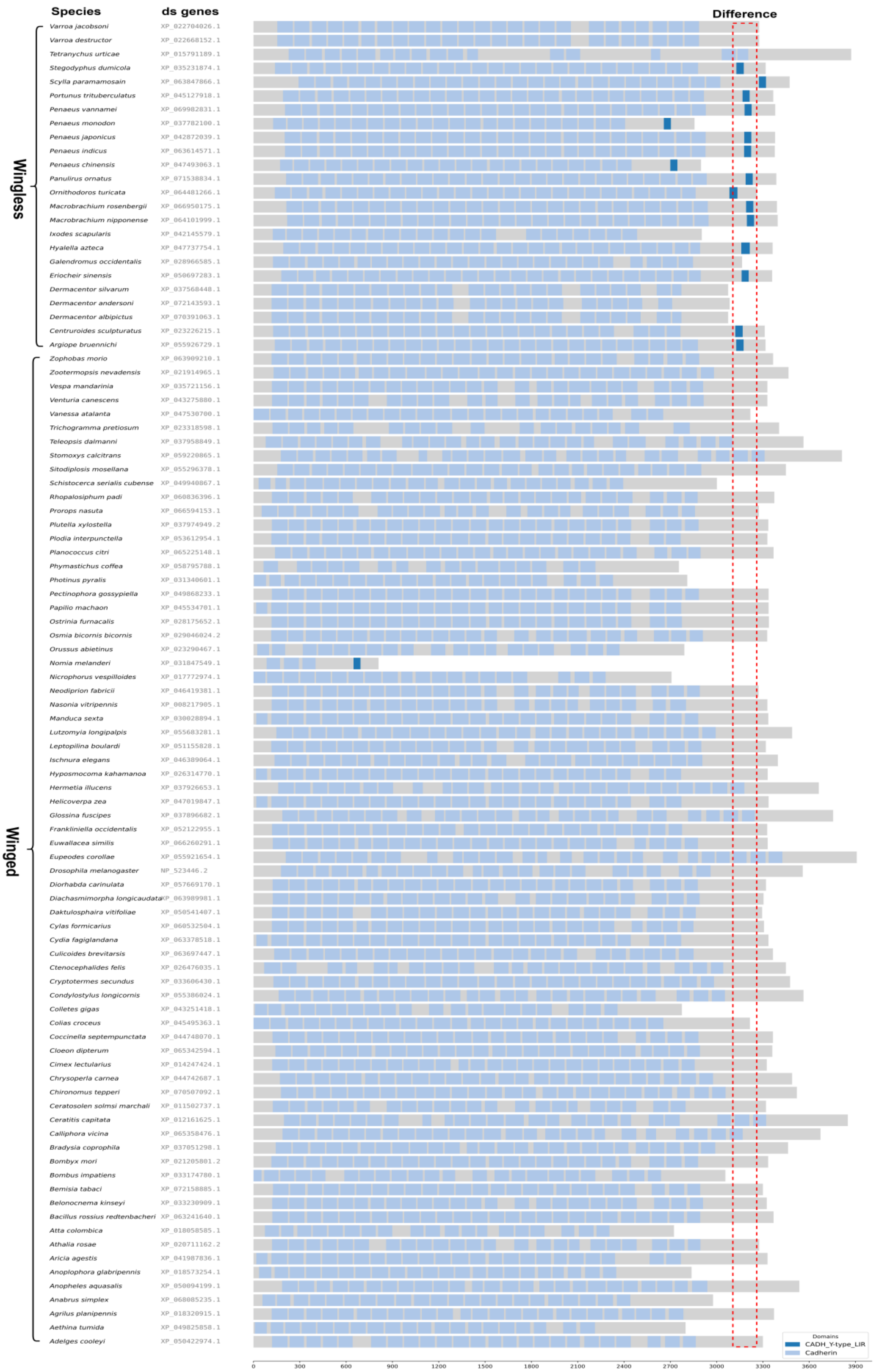

Figure S3. Domain difference of *ds* genes in wingless and winged insects.

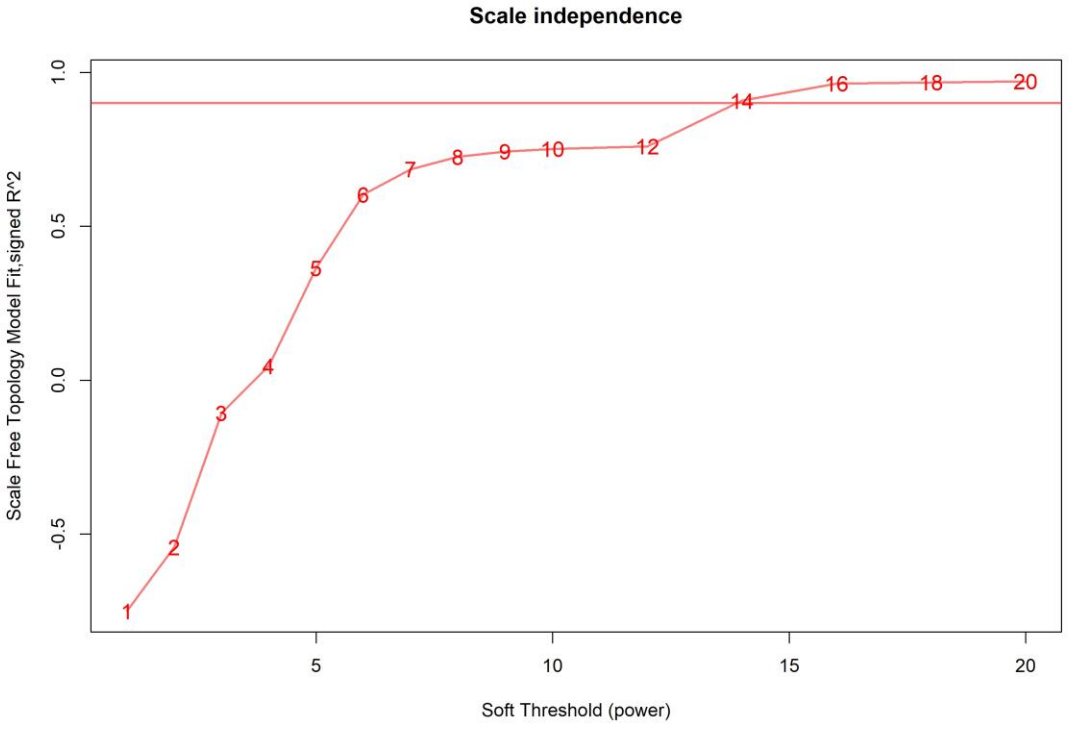

Figure S4. Scale-free fitting exponent for soft threshold power.

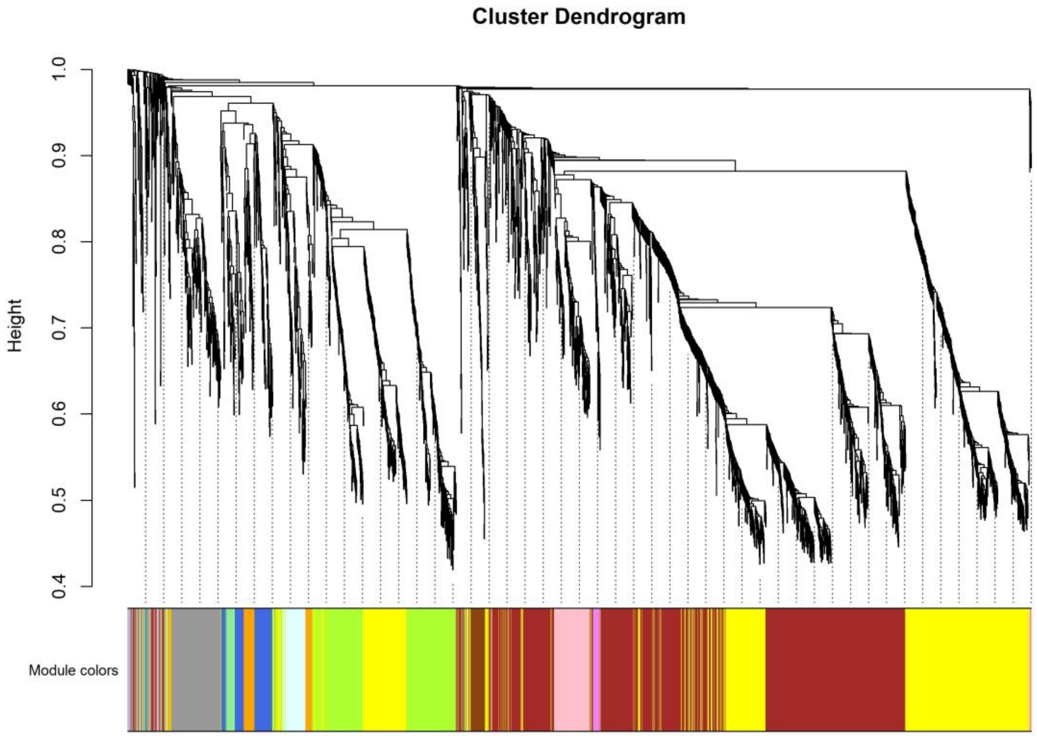

Figure S5. Gene and module color dendrogram. The y-axis shows the distance between clusters.

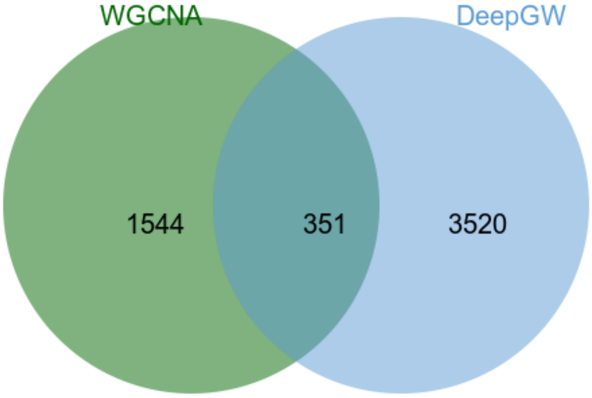

Figure S6. Number of shared genes identified by WGCNA and DeepGW.

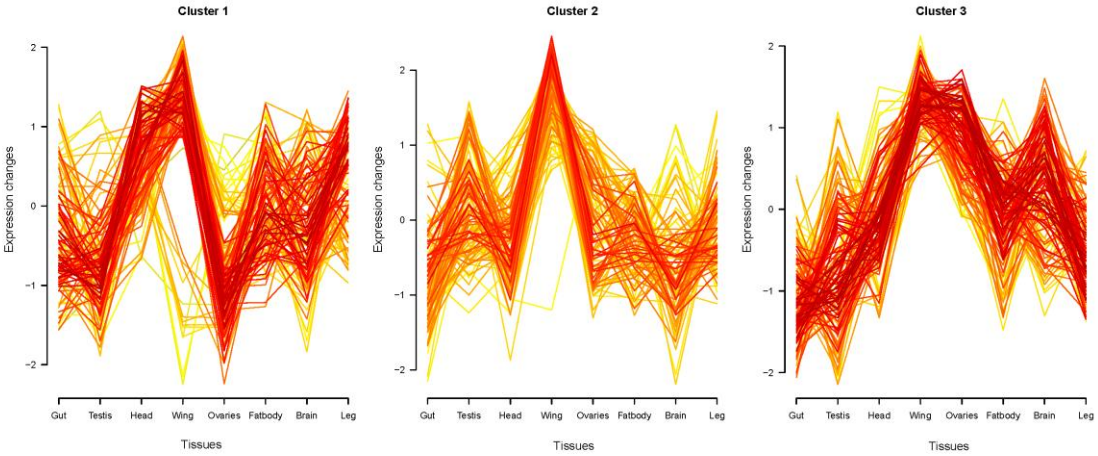

Figure S7. Expression patterns of 351 key genes in different tissues of *D. melanogaster*. Expression profiles of 351 key genes were clustered using the Mfuzz soft clustering algorithm. Expression values were log2(TPM + 1) and standardized prior to clustering. The fuzzification parameter (m) was estimated using the mestimate () function, and genes were grouped into three expression modules. Genes with membership values ≥ 0.7 were defined as core members of each cluster. Each line represents the normalized expression trajectory of an individual gene across tissues, and color gradients represent the membership strength of genes within clusters.

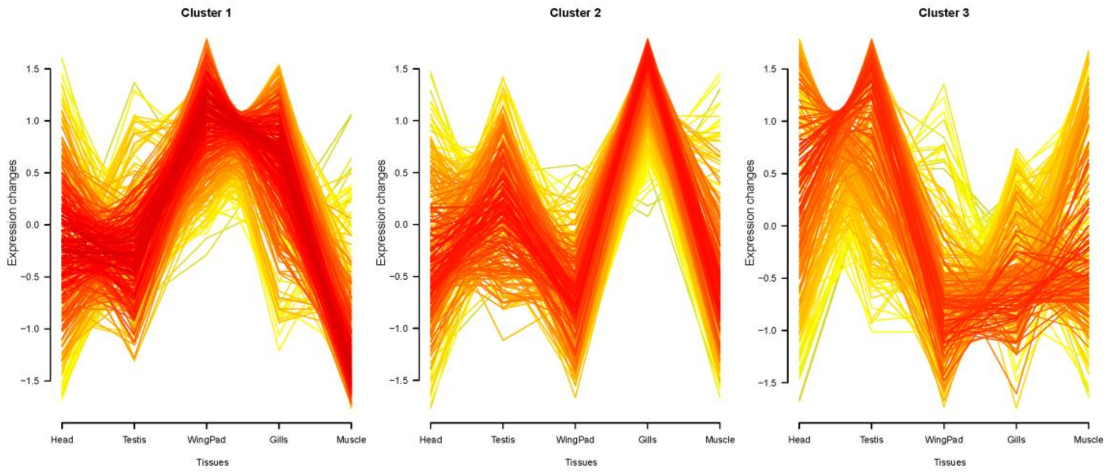

Figure S8. Expression patterns of orthologs for 351 key genes across different tissues in *C. dipterum*. Expression profiles of 351 key genes and their orthologs were clustered using the Mfuzz soft clustering algorithm. Expression values were log2(TPM + 1) and standardized prior to clustering. The fuzzification parameter (m) was estimated using the mestimate () function, and genes were grouped into three expression modules. Genes with membership values ≥ 0.7 were defined as core members of each cluster. Each line represents the normalized expression trajectory of an individual gene across tissues, and color gradients represent the membership strength of genes within clusters.

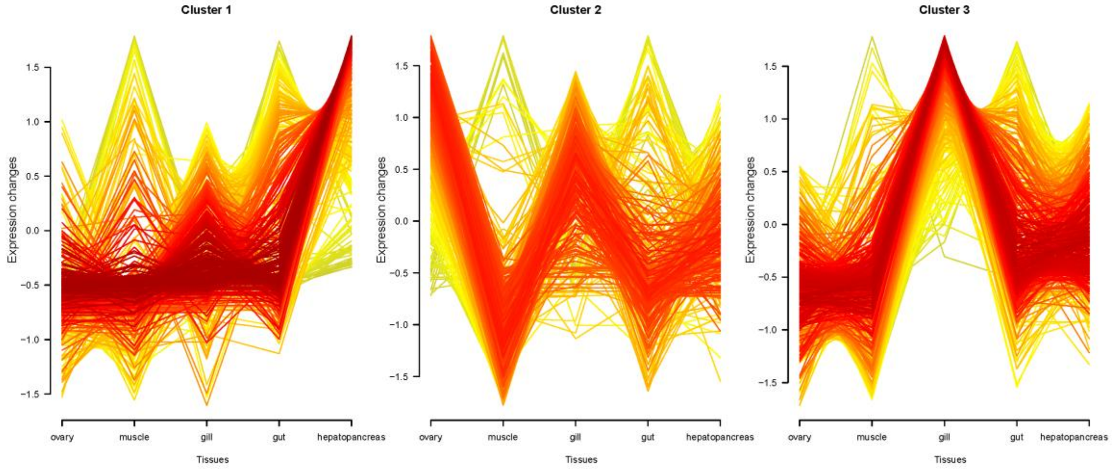

Figure S9. Expression patterns of orthologs for 351 key genes across different tissues in *P. vannamei*. Expression profiles of 351 key genes and their orthologs homologs were clustered using the Mfuzz soft clustering algorithm. Expression values were log2(TPM + 1) and standardized prior to clustering. The fuzzification parameter (m) wass estimated using the mestimate () function, and genes were grouped into three expression modules. Genes with membership values ≥ 0.7 were defined as core members of each cluster. Each line represents the normalized expression trajectory of an individual gene across tissues, and color gradients represent the membership strength of genes within clusters.
